# Differentiation between high-grade gliomas and solitary brain metastases based on multidiffusion MRI model quantitative analysis

**DOI:** 10.3389/fonc.2024.1401748

**Published:** 2024-10-14

**Authors:** Libing He, Meining Chen, Hongjian Li, Xiran Shi, Zhiqiang Qiu, Xiaoxue Xu

**Affiliations:** ^1^ Department of Radiology, Affiliated Hospital of North Sichuan Medical College, Nanchong, China; ^2^ MRI Research Institute, Huaxi MR Research Center (HMRRC), Chengdu, Sichuan, China; ^3^ Department of Radiology, Affiliated Hospital of North Sichuan Medical College, Nanchong, Sichuan, China

**Keywords:** glioma, brain metastasis, magnetic resonance imaging, neurite orientation dispersion and density imaging (NODDI), mean apparent propagator magnetic resonance imaging (MAP-MRI), diffusion magnetic resonance imaging (dMRI)

## Abstract

**Background and purpose:**

Differentiating high-grade gliomas (HGGs) from solitary brain metastases (SBMs) using conventional magnetic resonance imaging (MRI) remains challenging due to their similar imaging features. This study aimed to evaluate the diagnostic performance of advanced diffusion models, such as neurite orientation dispersion and density imaging (NODDI) and mean apparent propagator magnetic resonance imaging (MAP-MRI), incomparison to traditional techniques like diffusion-weighted imaging (DWI), diffusion tensor imaging (DTI), and diffusion kurtosis imaging (DKI) for distinguishing HGGs from SBMs.

**Methods:**

In total, 17 patients with HGGs and 26 patients with SBMs were prospectively recruited based on the established inclusion and exclusion criteria. Structural MRI sequences and diffusion spectrum imaging (DSI) were utilized to assess quantitative parameter models, including NODDI, MAP-MRI, DWI, DTI, and DKI. Quantitative parameters were measured for both the tumor parenchymal area and the peritumoral edema area. The quantitative parameters of the two patient groups were compared using either the independent Student’s *t*-test or the Mann–Whitney *U* test. The effectiveness of each model was evaluated using receiver operating characteristic (ROC) curves and calculating the area under the curve (AUC). Finally, the DeLong test was employed to compare the diagnostic performance of each model through pairwise comparisons of ROC curves.

**Results:**

Isotropic volume fraction (V_iso_) based on NODDI; mean squared displacement (MSD) and the return to plane probabilities (RTPP) based on MAP-MRI; radial diffusivity (RD_k_) and mean diffusivity (MD_k_) based on DKI; and axial diffusivity (AD), radial diffusivity (RD), and mean diffusivity (MD) based on DTI of the peritumoral edema tumor were significantly different between HGGs and SBMs (*p* < 0.05). The optimal single discriminant parameters for each model are NODDI_V_iso_, MAP-MRI_MSD, DKI_MD_k_, and DTI_AD. Among these, the AUC of V_iso_ (0.809) exceeds that of MSD (0.733), MD_k_ (0.718), and AD (0.779). The combined model, which incorporates DTI_AD, DKI_RD, and NODDI_V_iso_, demonstrated superior diagnostic performance (0.897).

**Conclusions:**

Advanced diffusion MRI quantitative parameters derived from NODDI, such as V_iso_, have the potential to enhance the differentiation between HGGs and SBMs. The integrated utilization of these models is anticipated to enhance diagnostic accuracy and refine MRI protocols for brain tumor assessment.

## Introduction

Gliomas, the most malignant subtype of neuroepithelial tumors, represent the predominant incidence of primary brain tumors (1). According to the latest Fifth edition of the World Health Organization (WHO) classification of central nervous system (CNS) tumors, gliomas are stratified into four grades (2). High-grade gliomas (HGGs), encompassing WHO grades III and IV, entail a grim prognosis, with a 5-year survival rate ranging from 25.9% to 49.4% for grade III and a mere 4.7% for grade IV (3). Brain metastases are prevalent malignant neoplasms in adults (4), occurring 10 times more frequently than primary brain malignancies (5). In certain instances, patients presenting multiple cerebral lesions with a history of primary malignancy may undergo a straightforward diagnosis of brain metastases. Nonetheless, solitary brain metastases (SBMs) manifest initially in nearly 30% of patients with systemic malignancies (6), potentially serving as the inaugural symptom of undiagnosed extracranial malignant tumors. Additionally, gliomas may manifest in patients with systemic cancer. Given the differences in medical staging, clinical management, and prognosis between HGGs and SBMs, accurately distinguishing these two types of malignant tumors is of great value for clinical decision-making.

Currently, magnetic resonance imaging (MRI) stands as the premier noninvasive modality for diagnosing intracranial tumors. Accurately distinguishing between HGGs and SBMs poses a challenge when patients present with isolated and markedly heterogeneously enhanced brain lesions, as both commonly display akin imaging features and enhancement patterns—including cysts, necrosis, circular enhancement, and peritumoral edema—on conventional MRI. This often leads to misdiagnoses in over 40% of cases (7). In such scenarios, while postoperative histopathological biopsy remains the gold standard for differentiation, it is associated with certain limitations, including surgical complications or infeasibility due to patients’ poor physical condition, tumor invasiveness, or proximity to critical brain regions. Consequently, resorting to noninvasive techniques becomes imperative for distinguishing between HGGs and SBMs (8, 9).

Many previous studies have explored various methodologies to tackle this issue, including MR perfusion-weighted imaging (PWI) (10–12), magnetic resonance spectroscopy (MRS) (10, 12), diffusion-weighted imaging (DWI) (13, 14), and diffusion tensor imaging (DTI) (10, 15). Notably, among these techniques, the DWI-based apparent diffusion coefficient (ADC) and DTI-based fractional anisotropy (FA) values are the most widely utilized metrics. However, there are controversial results regarding the ability of ADC and FA to distinguish between HGGs and SBMs.

In recent years, traditional MRI technology has increasingly struggled to meet the practical needs of clinical work. However, innovative diffusion MRI techniques, such as neurite orientation dispersion and density imaging (NODDI) (16) and the diffusion spectrum imaging (DSI)-based mean apparent propagator (MAP)-MRI (17), have demonstrated enhanced sensitivity in detecting changes in the microstructure of brain tissue. Promising results have been obtained using NODDI and MAP-MRI in multiple sclerosis (18), glioma grading (19), and Alzheimer’s disease (20). However, it remains uncertain whether these techniques outperform the more commonly used non-Gaussian-based diffusion kurtosis imaging (DKI) and Gaussian diffusion models such as DTI and DWI in differentiation between HGGs and SBMs. Therefore, this study aims to evaluate the effectiveness of NODDI, MAP-MRI, DKI, DTI, and DWI in distinguishing HGGs from SBMs.

## Materials and methods

### Study design and participants

**Figure 1:**
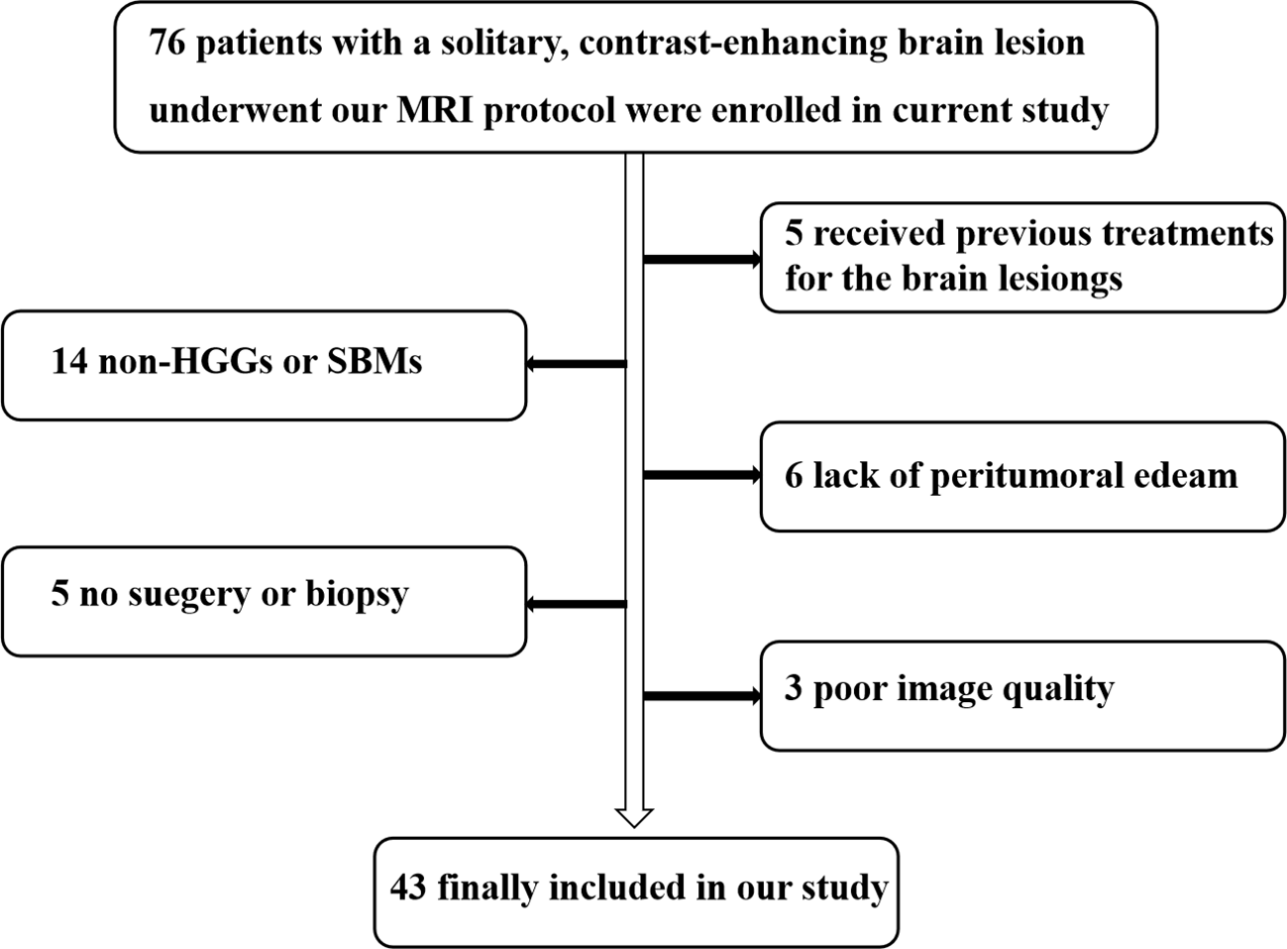
Flowchart illustrating the selection of the study population. HGGs, high-grade gliomas; SBMs, solitary brain metastases.

This study adhered to the principles outlined in the Helsinki Declaration and received approval from the ethics review committee of our institution. All participants provided written informed consent prior to undergoing MRI examinations. From December 2022 to November 2023, we recruited 76 patients suspected of having brain tumors based on MRI findings or other clinical assessments. The inclusion criteria were as follows: (1) patients with isolated brain lesions exhibiting significant, uneven enhancement on structural MRI; (2) patients diagnosed with HGGs or SBMs via postoperative pathological biopsy or stereotactic biopsy, in accordance with the 2016 WHO classification criteria for brain tumors; and (3) some SBM patients who were not subjected to surgical or pathological biopsy were clinically confirmed through follow-up (confirmation criteria included: histopathologically confirmed malignant tumors elsewhere in the body, with or without metastasis, and a significant reduction in intracranial lesions following radiotherapy and chemotherapy). The exclusion criteria were as follows: (1) patients with other brain tumors confirmed via histopathology that were not HGGs or SBMs; (2) insufficient peritumoral edema for further analysis; (3) any pretreatment of brain lesions prior to the MRI examination; and (4) poor MRI image quality due to motion artifacts. Ultimately, our study included 43 patients (27 men and 16 women, with an average age of 57.64 years and an age range of 17– 84 years). The selection process for research subjects is depicted in **Figure 1**.

### MRI protocols

In this study, all participants underwent MRI using a 3-T scanner (MAGNETOM Skyra, Siemens Healthineers, Erlangen, Germany) with a 32-channel head coil. Patients were positioned in a standard supine orientation for structural and diffusion-weighted MRI scans. The structural MRI protocol comprised axial T1-weighted images (T1WI), axial T2-weighted images (T2WI), axial DWI images, and axial contrast-enhanced 3D T1WI. The diffusion-weighted imaging protocol involved axial DSI conducted prior to the injection of the contrast agent, capturing 99 diffusion directions and 11 *b*-values (specifically, 0 s/mm^2^, 350 s/mm^2^, 650 s/mm^2^, 950 s/mm^2^, 1,000 s/mm^2^, 1,350 s/mm^2^, 1,650 s/mm^2^, 1,700 s/mm^2^, 2,000 s/mm^2^, 2,700 s/mm^2^, and 3,000 s/mm^2^) over a duration of 12 min. **Table 1** provides details on the specific parameters for each MRI sequence. The imaging coverage was designed to encompass the entire brain comprehensively, maintaining consistency across all axial sequences. The orientation of the scanning plane was aligned parallel to the anterior commissure–posterior commissure (AC-PC) line.

**Table 1:** Imaging sequences and acquisition parameters of structural and diffusion MRI.

Parameters	T1WI	T2WI	DWI	3D T1WI	DSI
TR (ms)	2,020	9,000	5,160	2,300	6,700
TE (ms)	17	93	61	2.34	108
TA	1 min 33 s	2 min 24 s	1 min 59 s	3 min 05 s	12 min
FOV (mm^2^)	220 × 220	220 × 220	220 × 220	220 × 220	250 × 250
Thickness (mm)	5	5	5	3	2
Slices	26	26	26	48	68
*b*-value (s/mm^2^)	–	–	0–1,000	–	0–3,000

T1WI, T1-weighted imaging; T2WI, T2-weighted imaging; DWI, diffusion weighted imaging; DSI, diffusion spectrum imaging; TR, time of repeatation; TE, time of echo; TA, scan time; FOV, field of view.

### Reconstruction and segmentation

All DSI data, initially stored in DICOM format, were converted to NIfTI format utilizing the DCM2NII tool. Subsequently, diffusion parameter models of DTI, DKI, MAP-MRI, and NODDI were reconstructed using in-house postprocessing software (NeuDiLab), which is based on the Diffusion Imaging in Python (DIPY) framework (https://dipy.org). The Elastix software (http://Elastix.isi.uu.nl/) was used to register all diffusion parameter models with axial contrast-enhanced T1W images, ensuring alignment within the same imaging space. For the registration of diffusion images, baseline data with contrast-enhanced T1W images were used. The quantitative analysis was conducted by a radiologist with 3 years of experience in neuroradiology, under the mentorship of a senior radiologist with 10 years of clinical neuroradiology experience in MRI, for the delineation of the regions of interest (ROIs Both radiologists were blind to the histological findings. Discrepancies were resolved through consensus. The ITK-SNAP (www.itk-snap.org) was used for the manual delineation of ROIs on contrast-enhanced T1WI and T2WI images. In identifying the ROIs, the area of significant enhancement within the maximum cross-sectional area of the tumor parenchyma was marked as the contrast-enhancing tumor ROIs, while the high-signal area on T2WI without enhancement was designated as the peritumoral edema ROIs. Regions suspected of containing necrotic, calcified, or cystic components were manually excluded. Ultimately, the delineated ROIs were transferred to the corresponding registered diffusion parameter model diagrams for the same patient using ITK-SNAP software, facilitating the calculation of average quantitative parameters for both the contrast-enhancing tumor area and peritumoral edema areas.

### Statistical analysis

The diffusion parameters of contrast-enhancing tumors and peritumoral edema were quantified. The Shapiro–Wilk test and the Levene *F*-test were used to assess normality and homogeneity of variance across all diffusion metrics. To evaluate differences in individual diffusion parameters, either an independent Student’s *t*-test or the Mann–Whitney *U* test was utilized. Furthermore, quantitative indicators showing significant statistical differences between HGGs and SBMs were integrated and analyzed using multiple logistic regression to develop a combined model. The performance of each parameter and the combined models was evaluated using the receiver operating characteristic (ROC) and the corresponding area under the ROC curve (AUC). Statistical metrics, including the calculation of the best threshold, specificity, sensitivity, AUC, and the 95% confidence interval (CI), were conducted using SPSS software (version 26.0, IBM, Armonk, NY, USA) to determine their efficacy in distinguishing between HGGs and SBMs. Pairwise comparisons of the ROC curves were also executed using the DeLong test. A two-tailed *p* < 0.05 was considered statistically significant.

## Results

### Characteristics of the study participants

Among the 43 enrolled patients, 17 (10 men and seven women; mean age, 58.11 years; age, 17–84 years) were confirmed to have HGGs, while 26 (17 men and nine women; mean age, 57.07 years; age, 26–73 years) were diagnosed with SBM based on histopathological examination or clinical follow-up. The primary sites of SBMs included 15 patients with lung carcinoma, three with breast cancer, two with ovarian cancer, one with esophageal cancer, one with gastric cancer, one with colon cancer, one with renal cancer, one with nasopharyngeal carcinoma, and one with atrial lymphoma.

### Comparison of diffusion parameters between HGGs and SBMs groups

**Figure 2:**
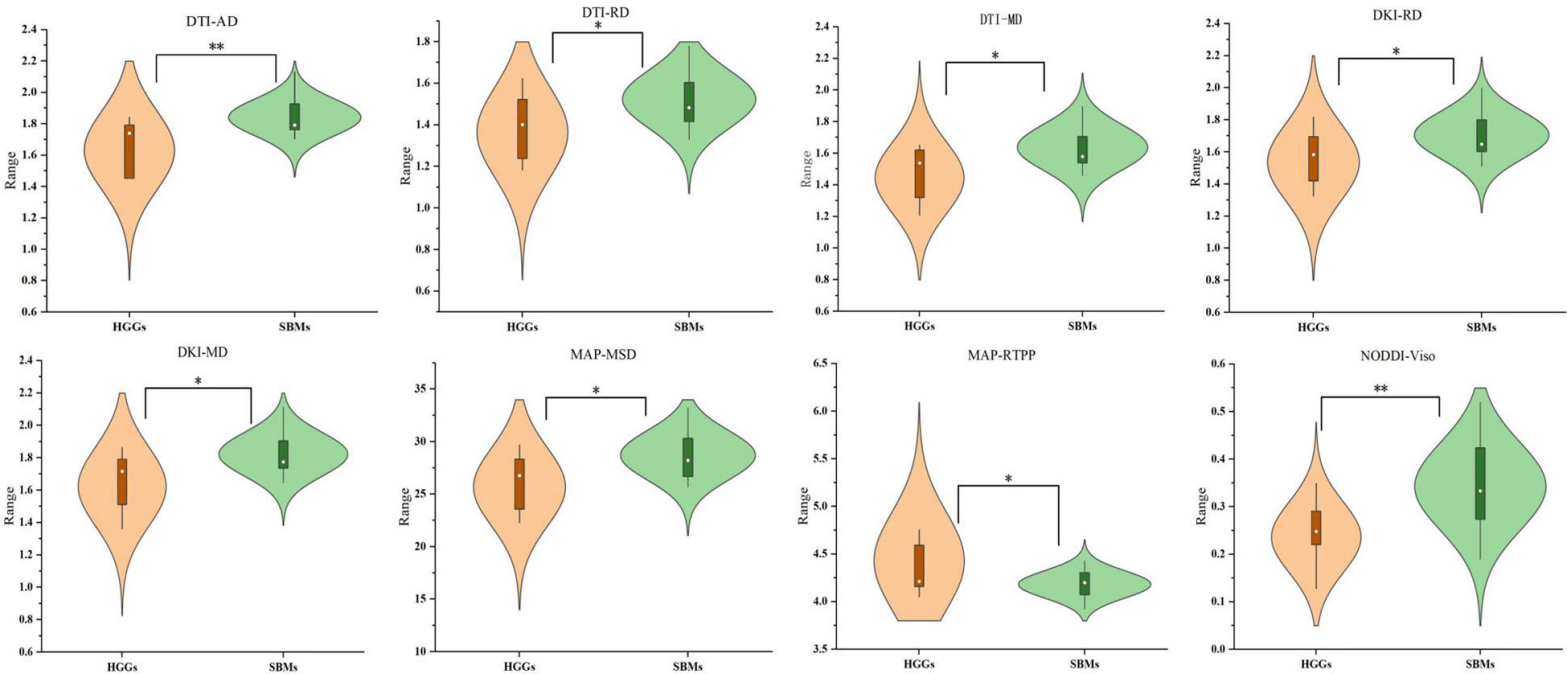
Violin diagram of significant diffusion parameters in peritumoral edema. ^*^ *p* < 0.05; ^**^ *p* < 0.01.

The mappings of diffusion parameters, including DWI_ADC, DTI_AD, DTI_RD, DTI_MD, DTI_FA, DKI_AK, DKI_RK, DKI_MK, DKI_AD, DKI_RD, DKI_MD, DKI_FA, MAP_NG, MAP_NGax, MAP_NGrad, MAP_MSD, MAP_QIV, MAP_RTOP, MAP_RTAP, MAP_RTPP, NODDI_Vic, NODDI_Viso, and NODDI_ODI, from one patient with HGG and one with SBM are shown in **Table 2**. As shown in **Table 2**, DTI_AD, DTI_RD, DTI_MD, DKI_RD, DKI_MD, MAP_MSD, and NODDI_V_iso_ of the peritumoral edema were significantly lower in the HGGs compared to SBMs (*p* = 0.004, *p* = 0.030, *p* = 0.026, *p* = 0.044, *p* = 0.026, *p* = 0.017, and *p* = 0.001, respectively). In contrast, MAP_RTPP of the peritumoral edema was significantly higher in the HGGs than in the SBMs (*p* = 0.039). No obvious differences were found among all other diffusion parameters in the contrast-enhancing tumors or peritumoral edema between the two groups (*p* > 0.05). **Figure 2** shows the violin diagram of the significant diffusion parameters in peritumoral edema.

**Table 2:** All diffusion parameters in the contrast-enhancing tumor or peritumoral edema between HGGs and SBMs.

Parameters	Contrast-enhancing tumor		*p*-value	Peritumoral edema		*p*-value
	HGGs (*n* = 17)	SBMs (*n* = 26)		HGGs (*n* = 17)	SBMs (*n* = 26)	
DWI						
ADC	1.168 ± 0.037	1.188 ± 0.052	0.951	1.430 ± 0.032	1.493 ± 0.021	0.095
DTI						
AD	1.183 ± 0.058	1.368 ± 0.069	0.477	1.631 ± 0.066	1.841 ± 0.025	0.004^**^
RD	1.162 ± 0.054	1.182 ± 0.056	0.806	1.364 ± 0.056	1.523 ± 0.030	0.030^*^
MD	1.236 ± 0.054	1.256 ± 0.067	0.688	1.045 ± 0.059	1.638 ± 0.031	0.026^*^
FA	0.125 ± 0.015	0.104 ± 0.008	0.293	0.140 ± 0.007	0.133 ± 0.007	0.531
DKI						
AK	0.620 ± 0.046	0.672 ± 0.044	0.386	0.453 ± 0.031	0.409 ± 0.010	0.338
RK	0.673 ± 0.042	0.708 ± 0.043	0.571	0.571 ± 0.028	0.520 ± 0.014	0.421
MK	0.647 ± 0.043	0.688 ± 0.044	0.599	0.506 ± 0.028	0.467 ± 0.012	0.496
AD_k_	1.565 ± 0.066	1.568 ± 0.083	0.734	1.857 ± 0.063	1.936 ± 0.086	0.101
RD_k_	1.313 ± 0.062	1.334 ± 0.065	0.821	1.536 ± 0.062	1.705 ± 0.032	0.044^*^
MD_k_	1.397 ± 0.062	1.411 ± 0.070	0.891	1.622 ± 0.063	1.822 ± 0.029	0.026^*^
FA_k_	0.183 ± 0.013	0.160 ± 0.007	0.089	0.192 ± 0.008	0.178 ± 0.006	0.164
MAP-MRI						
NG	0.157 ± 0.013	0.169 ± 0.012	0.536	0.118 ± 0.008	0.106 ± 0.004	0.404
NG_Ax_	0.132 ± 0.010	0.145 ± 0.009	0.347	0.101 ± 0.007	0.090 ± 0.003	0.421
NG_Rad_	0.084 ± 0.008	0.094 ± 0.007	0.386	0.059 ± 0.005	0.052 ± 0.002	0.370
MSD	22.214 ± 0.957	22.267 ± 1.063	0.972	25.684 ± 0.966	28.710 ± 0.506	0.017^*^
QIV	62.694 ± 5.931	60.510 ± 8.418	0.353	110.440 ± 9.572	128.436 ± 7.234	0.136
RTOP	2.268 ± 0.318	2.547 ± 0.359	0.496	1.476 ± 0.202	1.149 ± 0.042	0.122
RTAP	3.565 ± 0.290	3.659 ± 0.313	0.710	2.784 ± 0.212	2.422 ± 0.064	0.164
RTPP	5.074 ± 0.193	5.188 ± 0.228	0.421	4.428 ± 0.132	4.183 ± 0.031	0.039^*^
NODDI						
V_ic_	0.294 ± 0.034	0.343 ± 0.036	0.421	0.177 ± 0.023	0.151 ± 0.008	0.421
V_iso_	0.187 ± 0.022	0.239 ± 0.027	0.164	0.236 ± 0.019	0.343 ± 0.020	0.001**
ODI	0.456 ± 0.042	0.551 ± 0.039	0.111	0.246 ± 0.025	0.214 ± 0.015	0.155

All data are represented as mean ± standard deviation. ^*^ *p* < 0.05; ^**^ *p* < 0.01. FA, AK, RK, MK, FAk, NG, NG_Ax_, NG_Rad_, V_ic_, V_iso_, and ODI are dimensionless. The units of ADC, AD, RD, MD, AD_k_, RD_k_, and MD_k_ are 10{sp}−3{/sp} mm^2^/s; MSD (× 10^−5^ mm^2^), QIV (× 10^−10^ mm^5^), RTOP (× 10^5^ mm^−3^), RTAP (× 10^3^ mm^−2^), and RTPP (× 10^1^ mm^−1^).

### Distinguishing performance of diffusion parameters between HGGs and SBMs groups

**Figure 3:**
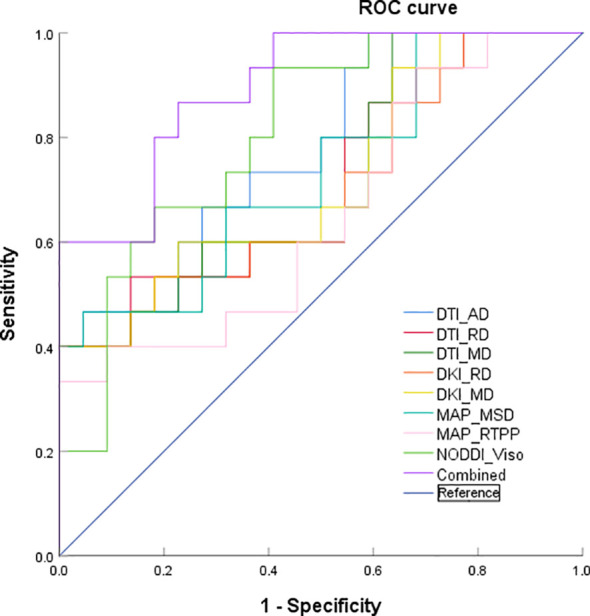
ROC curves of DTI_AD, DTI_RD, DTI_MD, DKI_RD, DKI_MD, MAP_MSD, MAP_RTPP, NODDI_V_iso_, and the combined model of the peritumoral edema.

**Figure 4:**
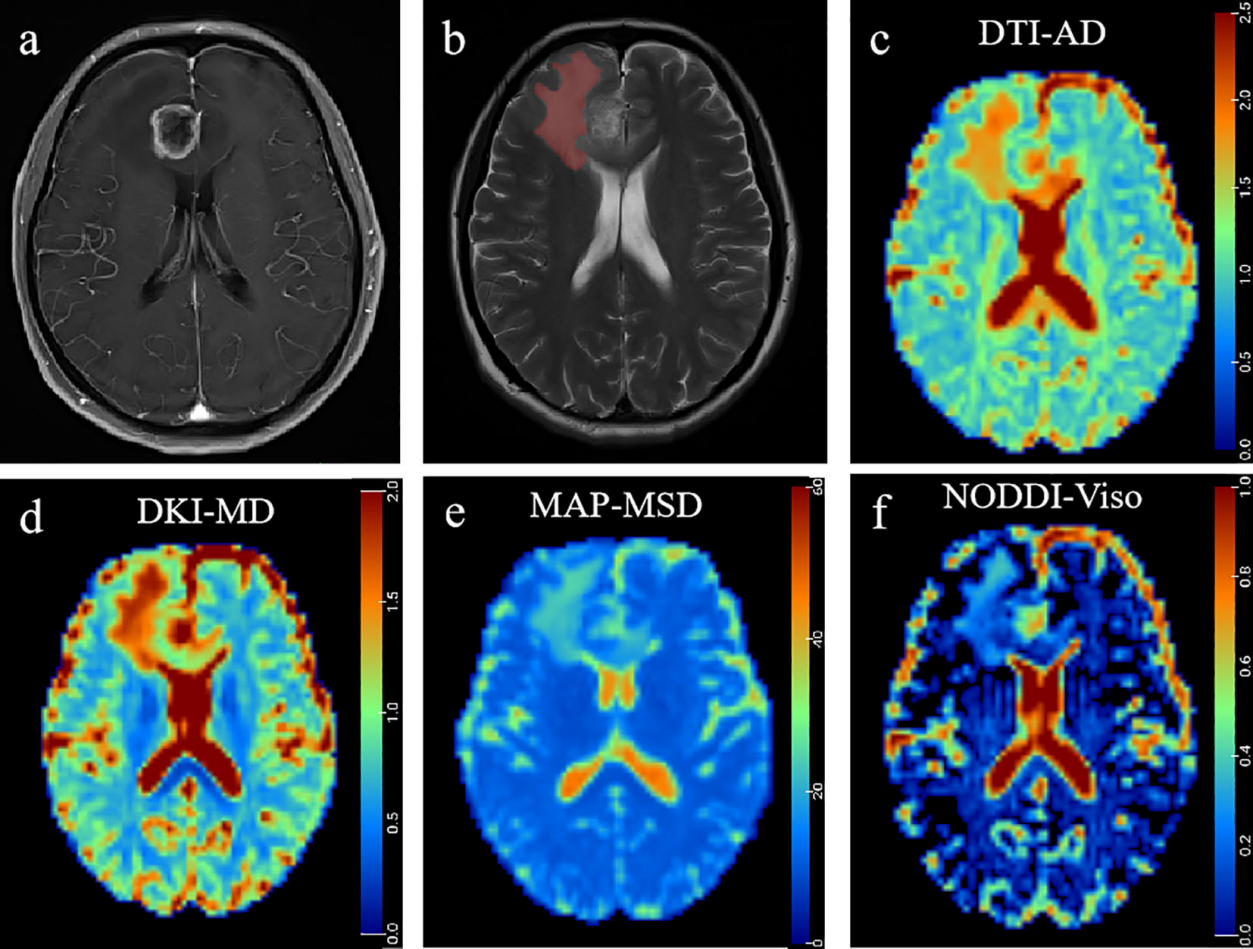
A 41-year-old man histopathologically confirmed to have isolated brain metastases from lung carcinoma. **(A)** Postcontrast T1W images and **(B)** T2W images represent an isolated contrast-enhancing tumor with peritumoral edema in the right frontal lobe. The red area represents the ROI of peritumoral edema. Pseudocolorful maps show the lesion AD **(C)**, MD_k_ **(D)**, MSD **(E)**, and V_iso_ **(F)** having a slight increase compared to the contralateral normal white matter.

**Figure 5:**
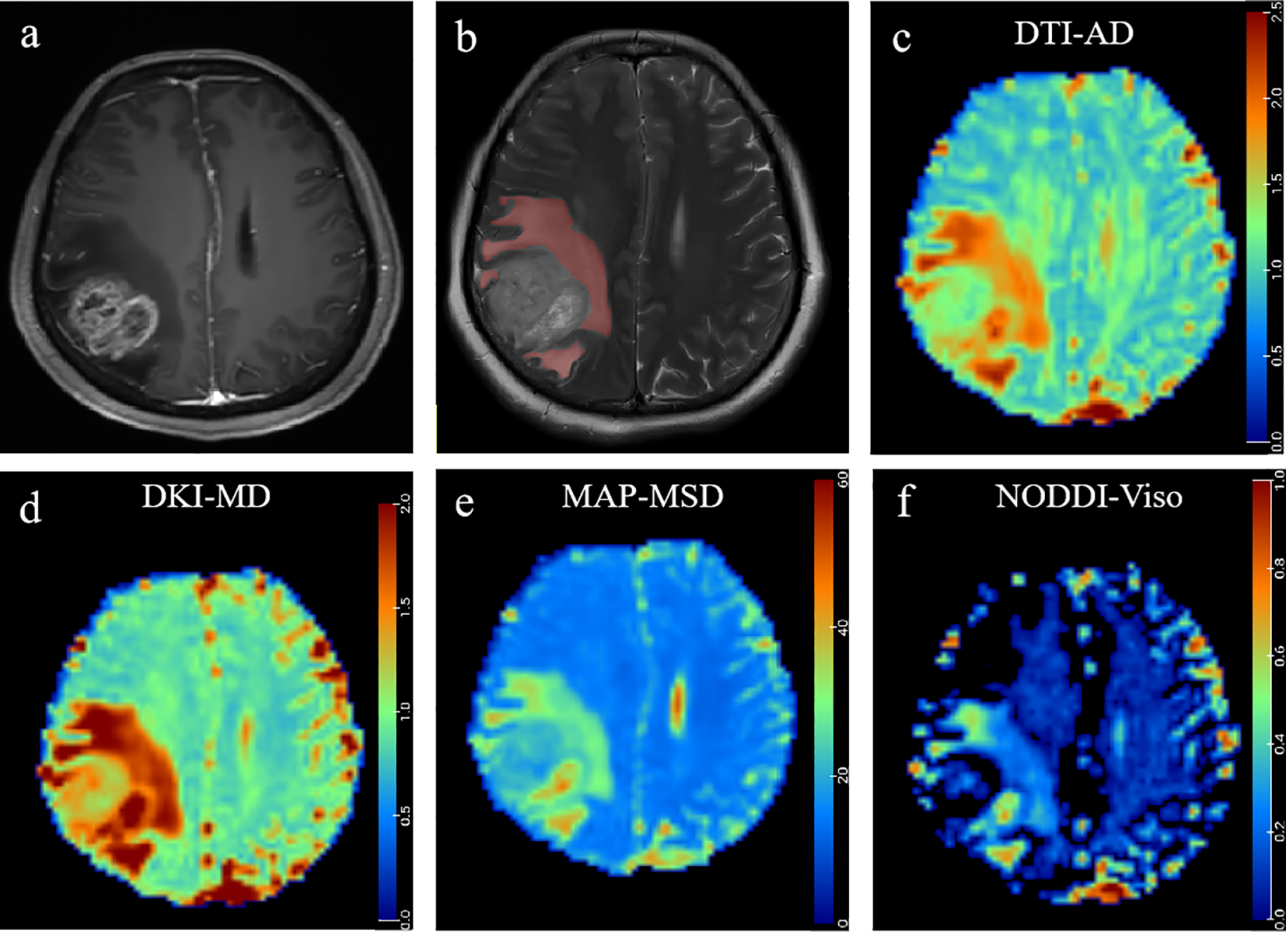
A 48-year-old woman histopathologically confirmed to have glioblastoma (WHO grade IV). **(A)** Postcontrast T1W images and **(B)** T2W images represent contrast-enhancing tumors with peritumoral edema in the right parietal occipital lobe. The red area represents the ROI of peritumoral edema. Pseudocolorful maps show the lesion AD **(C)**, MD_k_ **(D)**, MSD **(E)**, and V_iso_ **(F)** having a slight increase compared to the contralateral normal white matter.

ROC curve analyses of the significant diffusion parameters of the peritumoral edema are shown in **Table 3** and **Figure 3**. The best individual metrics for DTI, DKI, MAP-MRI, and NODDI were DTI_AD, DKI_MD, MAP_MSD, and NODDI_V_iso_, respectively (AUC = 0.779, 0.718, 0.733, and 0.809, respectively). Among these metrics, NODDI_V_iso_ had the highest AUC of 0.809, achieving 93.3% sensitivity and 59.9% specificity, with a 95% CI of 0.671 to 0.947, and the best cut-off value of 0.302 for distinguishing HGGs. The DeLong test was used for pairwise comparison of the ROC curves, revealing no significant differences between the parameters (all *p >* 0.05). When combined to create a multiple logistic regression analysis model, the combined model of DTI_AD, DKI_RD, and NODDI_V_iso_ showed better diagnostic efficacy (AUC: 0.897, 95% CI: 0.799 to 0.995, sensitivity: 86.7%, specificity: 77.3%) compared to any single parameter models, although this difference was not statistically significant. The typical cases of HGGs and SBMs are shown in **Figures 4**, **5**.

**Table 3:** Diagnostic performance for different MRI diffusion parameters.

Parameters	Cutoff value	AUC	Sensitivity	Specificity	95% CI
DTI_AD	1.713	0.779	46.7	95.5	0.629–0.929
DTI_RD	1.405	0.712	53.3	86.4	0.538–0.886
DTI_MD	1.658	0.718	66.7	36.4	0.547–0.890
DKI_RD	1.518	0.697	40.0	95.5	0.518–0.876
DKI_MD	1.731	0.718	60.0	77.3	0.546–0.891
MAP_MSD	26.104	0.733	46.7	95.5	0.566–0.901
MAP_RTPP	4.348	0.652	40.0	90.9	0.467–0.836
NODDI_V_iso_	0.302	0.809	93.3	59.1	0.671–0.947
The combined model	0.633	0.897	86.7	77.3	0.799–0.995

95% CI, 95% confidence interval.

## Discussion

This study used a 12-min diffusion sequence to investigate the ability of NODDI, MAP, DKI, DTI, and DWI to distinguish between GBM and SBM. All parameters of NODDI, MAP, DKI, DTI, and DWI were reconstructed using specialized postprocessing tools. Our results confirmed that HGGs and SBMs showed distinctive NODDI, MAP-MRI, DKI, and DTI-based diffusion parameters in the peritumoral edema region, while no differences were observed for any of the diffusion parameters in the contrast-enhancing tumor region. NODDI-based peritumoral V_iso_ is useful for distinguishing GBM and SBM, and the combination of DTI_AD, DKI_RD, and NODDI_V_iso_ further enhances classification accuracy.

Patients diagnosed with HGG or SBM exhibit divergent treatment pathways and survival rates, underscoring the critical importance of noninvasive and accurate differential diagnoses (21). Relying solely on conventional MRI techniques to differentiate between these malignancies poses considerable difficulties (7). Recent advancements in diffusion-weighted MRI technology have significantly enhanced our capability to detect microstructural alterations within brain tissue. Traditional Gaussian probability distribution models inadequately capture the intricate microanatomy of complex brain tissues. In contrast, non-Gaussian diffusion models, such as NODDI, MAP-MRI, and DKI offer a more accurate representation of water molecule dispersion, thereby more effectively reflecting the heterogeneity and complexity of the tissue microenvironment (22).

NODDI is an increasingly popular DWI technique with significant potential for clinical studies (23), as it provides a three-compartment microstructure model (intracellular, extracellular, and cerebrospinal fluid (CSF)) for each voxel. This approach can simultaneously describe the microstructural characteristics of dendrites and axons in both gray and white matter, providing more specific neuronal change data than standard DTI analysis (16). In our study, we demonstrated that NODDI-based V_iso_ probably outperformed other non-Gaussian or Gaussian diffusion metrics in differentiating between HGGs and SBMs. Although no single diffusion parameter can completely capture the complexity of neural tissue, our results suggest that V_iso_ may serve as a sensitive imaging biomarker in neurooncology research and warrants further investigation (8).

NODDI-V_iso_ represents isotropic Gaussian diffusion within the tissue. Caverzasi et al. (24) applied the NODDI model to evaluate various brain lesions. Unlike tumor-infiltrated edema, they found that qualitative results of NODDI color images can be used to distinguish vascular edema (e.g., brain metastases, lymphoma, and toxoplasmosis), which is characterized by a significant increase in V_iso_. Their findings are consistent with our results. Yoshihito et al. (25) applied the NODDI model to distinguish glioblastoma from brain metastases and found that the V_iso_ of the peritumoral edema was significantly lower in glioblastoma than in brain metastases. They suggested that the increased signal on the V_iso_ map is related to vascular edema. This result can be explained by the degradation of the extracellular matrix by acetyl heparanase and matrix metalloproteinases, which allows metastatic brain tumors to grow into the brain parenchyma in a dilated and noninfiltrating manner, resulting in a high degree of isotropy (26). Our data support their discovery and align with the hypotheses regarding vascular edema and invasive edema (27).

MAP-MRI is a novel magnetic resonance diffusion model based on DSI that more accurately reflects changes in the structure of white matter fiber bundles in the brain. Our study found that HGGs showed lower mean-squared displacement (MSD) and higher return-to-plane probabilities (RTPP) values than SBMs. MSD is a second-order displacement indicator of molecular diffusion distance and is more sensitive than the mean diffusivity (MD) in DTI (28). RTPP represents the probability of water molecules returning to the radial direction of the main diffusion direction (29). This phenomenon may occur because GBM tumor cells produce a large amount of specific extracellular matrix, mainly tenascin (30), which accumulates in the extracellular matrix and acts as a component of cell adhesion and migration (31). As a result, water molecules are more likely to return to their starting positions, leading to more restricted diffusion.

For DTI, we found that SBMs showed higher peritumoral axial diffusivity (AD), radial diffusivity (RD), and MD values than SBMs. MD reveals the rate of diffusion motion of water molecules, while AD and RD reflect the diffusion rates of water vertical and parallel scalars along the white matter tracts, respectively (32). MD, AD, and RD are all negatively correlated with the number of tumor cells (33, 34), which helps explain why metastatic lesions often form simple vascular edema around the tumor, resulting in lower cell density. As an extension of DTI, DKI-based RD and MD in our study showed similar change patterns with DTI-based RD and MD.

Prior research has used various diffusion metrics in efforts to distinguish tumor parenchymal areas between HGGs and SBMs; however, the findings remain controversial (8, 25, 35, 36). In the current study, despite utilizing five different diffusion models, no significant differences were observed in the diffusion parameters among the contrast-enhancing tumors of HGG and SBM. These results imply that diffusion metrics may not effectively discern the heterogeneity inherent to these distinct types of malignant tumors.

Our study encountered several limitations. Firstly, it was constrained by a relatively small sample size. Secondly, the majority of SBMs analyzed originated from lung cancer, suggesting potential deviations in results when assessing SBMs from other primary sites. Thirdly, the analysis was limited to mean diffusion metrics within ROIs, and it was noted that the tumor volume in HGG patients exceeded that in SBM patients. Lastly, this research did not incorporate other functional MR techniques, such as perfusion imaging and MR spectroscopy, which are essential for comprehensive comparative studies.

## Conclusions

Advanced diffusion MRI quantitative parameters derived from NODDI, such as V_iso_, have the potential to enhance the capability to differentiate between HGGs and SBMs. The integrated utilization of these models is anticipated to enhance diagnostic accuracy and refine MRI protocols for brain tumor assessment.

## Data Availability

The original contributions presented in the study are included in the article/supplementary material. Further inquiries can be directed to the corresponding author.
